# Intestinal intussusception and progressive anemia due to pyogenic granuloma of the ileum: a case report

**DOI:** 10.1186/s40792-021-01170-2

**Published:** 2021-04-07

**Authors:** Kodai Nagakari, Akikazu Yago, Yu Ohkura, Daisuke Tomita, Shusuke Haruta, Yutaka Takazawa, Masaki Ueno

**Affiliations:** Department of Gastroenterological Surgery, Toranomon Hospital, #311, 6-5-38 Akasaka, Minato-ku, Tokyo, 107-0052 Japan

**Keywords:** Pyogenic granuloma, Intestinal intussusception, Small bowel obstruction

## Abstract

**Background:**

Pyogenic granuloma is a benign vascular tumor, usually occurring on the skin or in the oral cavity. Small intestinal pyogenic granuloma is extremely rare, but intestinal intussusception due to the tumor is even rarer. Only 3 cases have been reported in the English literature at this writing.

**Case presentation:**

An 86-year-old woman presented with abdominal pain and vomiting. Laboratory data discovered anemia. Contrast-enhanced computed tomography revealed small bowel obstruction due to intestinal intussusception. After decompression by long tube for 1 week, the obstruction did not improve and the anemia got worse. Therefore, laparoscopic assisted small bowel resection was performed as a diagnostic therapy. Pathology confirmed the diagnosis of pyogenic granuloma. The postoperative course was uneventful and the patient was discharged 10 days after surgery.

**Conclusions:**

We experienced a case of intestinal intussusception and progressive anemia due to pyogenic granuloma of the ileum. Although the condition is extremely rare, surgeons must take into consideration the tumor in similar cases, and complete surgical resection is required.

## Background

Pyogenic granuloma (PG), also known as lobular capillary hemangioma, is a benign vascular tumor characterized by rapid progression and friable surface. Although it was first reported in 1897 by Poncet et al. by the name of Botryomycosis humaine [1], Hartzell [2] called it pyogenic granuloma in 1904. It usually occurs on the skin or in the oral cavity, and PGs in the small intestine are extremely rare. Small intestinal PGs are often detected during examination of obscure gastrointestinal bleeding. To our knowledge, only 44 cases were reported in the English literature. In most of the cases, surgery or EMR (endoscopic mucosal resection) is performed as diagnostic therapy [3, 4].

Intestinal intussusception in adults is uncommon. It is different from child intussusception in various aspects, and almost 90% of all cases are associated with pathologic lead points. Because of the significant risk of malignancy, surgical resection is generally recommended [5].

We herein describe a patient with intestinal intussusception and progressive anemia caused by PG in the ileum.

## Case presentation

**Fig. 1:**
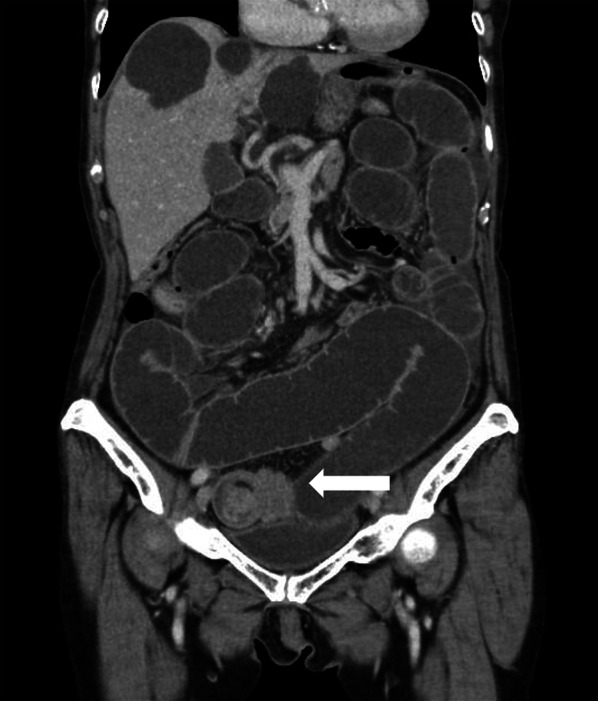
Late-phase contrast-enhanced CT. Target sign is detected in the small intestine in right lower abdomen (arrow), which indicates intussusception. The small intestine is diffusely dilated. No ascites or intestinal ischemic changes are seen

In May 2020, an 86-year-old woman presented to the emergency department with abdominal pain and vomiting. Contrast-enhanced computed tomography showed small bowel intussusception (Fig. 1) in right lower abdomen, and proximal bowel loops of the lesion were diffusely dilated. A slightly enhanced nodule was detected at the lead point of the intussusception, which made us suspect the existence of a tumor, such as gastrointestinal stromal tumor, small intestine cancer, neuroendocrine tumor, and leiomyoma. Laboratory examination was significant for iron deficiency anemia with hemoglobin level of 7.9 g/dL. We considered performing emergency laparotomy at this point, but did not, because the patient’s general condition was stable and intestinal necrosis was not suspected. We considered elective laparoscopic surgery after decompression of the small bowel is better. Hence, conservative treatment by long tube decompression was carried out. Although her general condition improved and the small bowel seemed to be decompressed by abdominal X-ray, the volume of drainage from the long tube remained high, constipation persisted and her hemoglobin level was dropped to 6.4 g/dL in 1 week. Therefore, we decided to perform diagnostic laparoscopy, after transfusion of red blood cells.

**Fig. 2:**
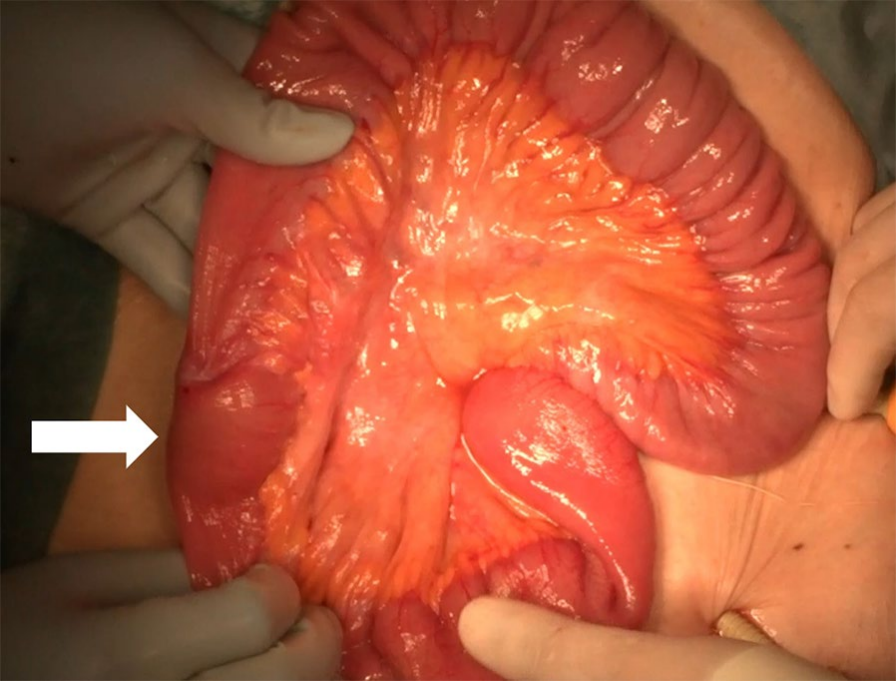
Intraoperative findings. A mass, approximately 4 cm in diameter, as a leading point for intestinal intussusception is detected 120 cm proximal to the Bauhin’s valve (arrow)

**Fig. 3:**
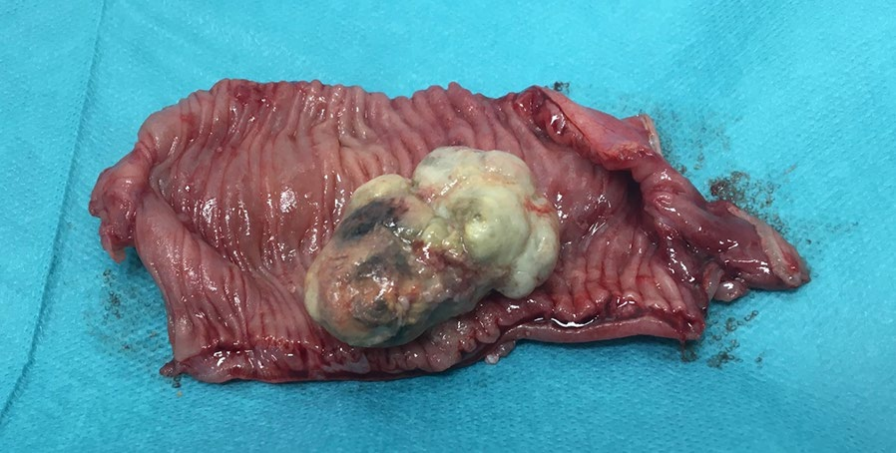
Resected specimen. The tumor originated from the mucosa of the ileum. The surface is whitish to grayish with erosion and inflammatory exudes

**Fig. 4:**
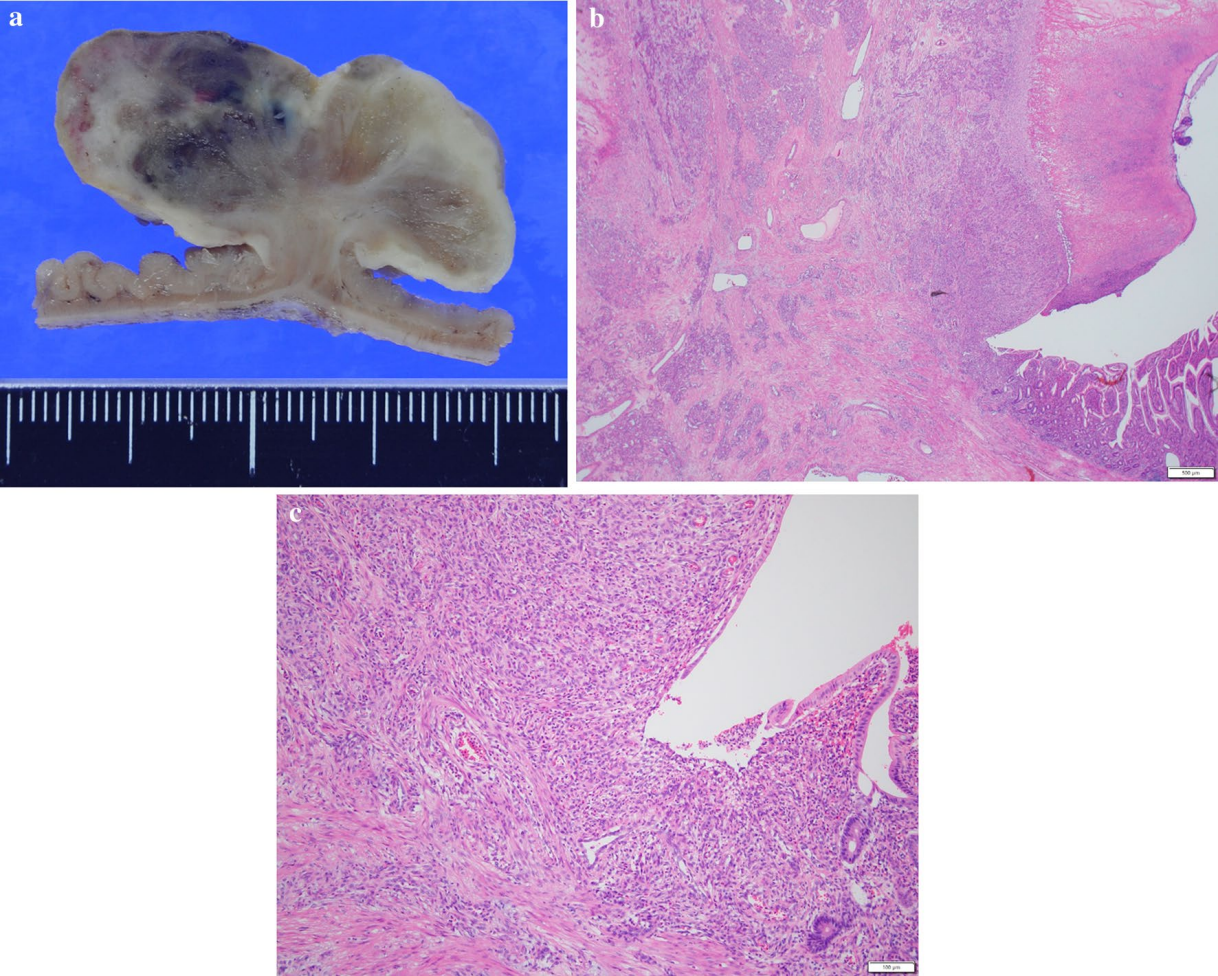
Histological examination of the tumor. Macroscopically, a milky-whitish to grayish solid tumor, 4.2 × 3.2 × 1.8 cm in size, forms a semipedunculated polypoid structure in the ileum (**a**). Microscopically, it consists of lobulated lesions with capillary proliferation infiltrated with inflammatory cells, accompanied with erosive mucosa on its surface (hematoxylin and eosin [H&E] stain) (**b**, **c**)

The operation was performed with 3 ports. Initially, a 12-mm trocar was inserted through an umbilical incision. Two trocars were then inserted in the left flank. After confirming the diagnosis of intussusception and absence of abdominal adhesions, a mini-laparotomy was made to observe whole small intestine. The lesion was easily identified as a mass, approximately 4 cm in diameter, had caused a small bowel intussusception (Fig. 2). Considering the possibility of malignancy, such as small intestinal adenocarcinoma, we decided to remove the mass with approximately 10 cm of proximal and distal margin. We did not perform lymph node dissection because the necessity of lymph node dissection for small intestinal adenocarcinoma is controversial, and lymph node metastasis was not suspected during the operation. The resected specimen showed a 4.2 × 3.2 × 1.8 cm grayish-white semipedunculated polyp at the center (Fig. 3). Histologically, it consisted of lobulated lesions with capillary proliferation infiltrated with inflammatory sells, accompanied with erosive mucosa on its surface (Fig. 4), consistent with pyogenic granuloma.

The postoperative course was uneventful. The long tube was removed 3 days after surgery and the patient was discharged 10 days after surgery. Her anemia has improved with the hemoglobin level of 9.3 g/dL.

## Discussion

PG is a capillary-proliferative neoplasm, which does not cause pain. It often exists as stalked or semipedunculated polyps, the color is generally dark black or bright red. The surface is frequently ulcerated. PGs occur on the skin or mucous membrane, and mucosal PGs usually occur in the oral cavity. They seldom develop in the digestive tract [3], however when they do, they can be a cause of gastrointestinal hemorrhage. Although the pathogenesis remains unknown, infection or irritation-induced granulation is suggested to be concerned, because 38–70.5% of patients are reported to have a history of infection or trauma. Many patients with PG in the oral cavity have experienced chronic irritation such as ill-fitting dental appliances, bites, or sharp teeth. In the gastrointestinal tract, mucosal damage from fish bones or exposure to gastric acid can be chronic irritation which generates PG; whereas, there are also some reports of PG which developed without any trauma or identified trigger (5). Histopathologically, PG is a polypoid hemangioma characterized by a lobular arrangement of capillaries at the base with enlarged vascular endothelial cells and inflammatory cell infiltration in the stroma.

PG in the small intestine is a rare entity. Our search of the PubMed database revealed only 44 reported cases of small intestinal PG [3]. Small intestinal PG is often found as a cause of obscure gastrointestinal bleeding, and capsule endoscopy is the modality that is most likely to effectively detect PG. Surgery or EMR are usually recommended as diagnostic therapies.

Adult intussusception is relatively rare, which occupies 5% of all cases of intussusception. As a cause of mechanical bowel obstructions in adults, it accounts for only 1–5%. The condition differs in many aspects from child intussusception. Child intussusception is generally primary and benign. 80% of the patients can be successfully treated via pneumatic or hydrostatic reduction of the intussusception. On the other hand, almost 90% of adult intussusception are due to a pathologic lead point, such as polyps, benign and malignant tumors, colonic diverticulum, Meckel’s diverticulum and postoperative adhesions, commonly discovered during operation [6]. Because of large proportion of structural anomalies and the high risk of malignancy, approximately 65% [7, 8], surgical resection is most frequently chosen as the treatment [9].

In our case, the patient was suffering from progressive anemia and small bowel obstruction due to intussusception. Although we considered performing capsule endoscopy or enteroscopy to make an accurate diagnosis, we decided not to, considering the patients situation. Instead, we performed small bowel resection as a diagnostic therapy and the pathology confirmed the diagnosis of PG. In our search of the PubMed database, 3 reported cases of small intestinal PG were associated with intussusception, and only 1 case was complicated by progressive anemia, which required transfusion [10–12]. Surgery was performed in all cases.

## Conclusions

In conclusion, we experienced a case of intestinal intussusception and progressive anemia due to PG of the ileum, which is extremely rare. To our knowledge, only 1 case has been reported in the English literature. Further studies involving more patients are needed.

## Data Availability

The dataset supporting the conclusions of this article is included within the article.

## Abbreviations

PG: Pyogenic granuloma
EMR: Endoscopic mucosal resection
